# Determining the Neural Substrate for Encoding a Memory of Human Pain and the Influence of Anxiety

**DOI:** 10.1523/JNEUROSCI.0750-17.2017

**Published:** 2017-12-06

**Authors:** Ming-Tsung Tseng, Yazhuo Kong, Falk Eippert, Irene Tracey

**Affiliations:** ^1^Graduate Institute of Brain and Mind Sciences, National Taiwan University College of Medicine, Taipei, 10051, Taiwan,; ^2^Oxford Center for Functional Magnetic Resonance Imaging of the Brain & Nuffield Division of Anaesthetics, Nuffield Department of Clinical Neurosciences, University of Oxford, Oxford, OX3 9DU, United Kingdom,; ^3^Institute of Psychology, Chinese Academy of Sciences, Beijing, 100101, China, and; ^4^Department of Psychology, University of Chinese Academy of Sciences, Beijing, 100049, China

**Keywords:** cognition, encoding, fMRI, pain, sensation, vibration

## Abstract

To convert a painful stimulus into a briefly maintainable construct when the painful stimulus is no longer accessible is essential to guide human behavior and avoid dangerous situations. Because of the aversive nature of pain, this encoding process might be influenced by emotional aspects and could thus vary across individuals, but we have yet to understand both the basic underlying neural mechanisms as well as potential interindividual differences. Using fMRI in combination with a delayed-discrimination task in healthy volunteers of both sexes, we discovered that brain regions involved in this working memory encoding process were dissociable according to whether the to-be-remembered stimulus was painful or not, with the medial thalamus and the rostral anterior cingulate cortex encoding painful and the primary somatosensory cortex encoding nonpainful stimuli. Encoding of painful stimuli furthermore significantly enhanced functional connectivity between the thalamus and medial prefrontal cortex (mPFC). With regards to emotional aspects influencing encoding processes, we observed that more anxious participants showed significant performance advantages when encoding painful stimuli. Importantly, only during the encoding of pain, the interindividual differences in anxiety were associated with the strength of coupling between medial thalamus and mPFC, which was furthermore related to activity in the amygdala. These results indicate not only that there is a distinct signature for the encoding of a painful experience in humans, but also that this encoding process involves a strong affective component.

**SIGNIFICANCE STATEMENT** To convert the sensation of pain into a briefly maintainable construct is essential to guide human behavior and avoid dangerous situations. Although this working memory encoding process is implicitly contained in the majority of studies, the underlying neural mechanisms remain unclear. Using fMRI in a delayed-discrimination task, we found that the encoding of pain engaged the activation of the medial thalamus and the functional connectivity between the thalamus and medial prefrontal cortex. These fMRI data were directly and indirectly related to participants' self-reported trait and state anxiety. Our findings indicate that the mechanisms responsible for the encoding of noxious stimuli differ from those for the encoding of innocuous stimuli, and that these mechanisms are shaped by an individual's anxiety levels.

## Introduction

To cope with the changing external environment, we temporarily maintain and manipulate sensory information to guide our actions. The ability to convert sensory stimuli, especially those that are painful, into a maintainable construct when the stimulus is no longer accessible is thus essential to human behavior. Different from bottom-up perceptual processing, evidence suggests distinct central processing mechanisms for this working memory encoding process (Palva et al., 2011; Bergmann et al., 2012). Indeed, this encoding process is implicitly contained in the majority of studies in which subjects are asked to encode somatosensory stimuli and then either give perceptual ratings or compare the original stimulus with another stimulus after a brief delay (Kong et al., 2006; Pleger et al., 2006; Preuschhof et al., 2006; Albanese et al., 2007; Kostopoulos et al., 2007; Oshiro et al., 2007; Lobanov et al., 2013). However, most experimental designs do not distinguish encoding-related brain activity from the response evoked by pure sensory stimulation. In contrast to the compelling evidence showing that distinct brain regions, such as hippocampus, are vital to transform information into constructs that can be recalled from long-term memory (Dolan and Fletcher, 1997), only little is known about the neural mechanisms underlying the working memory encoding of somatosensation.

Despite a few studies investigating working memory encoding of either painful stimuli (Kong et al., 2006; Albanese et al., 2007; Lobanov et al., 2013) or nonpainful somatosensory stimuli (Pleger et al., 2006; Preuschhof et al., 2006; Kostopoulos et al., 2007) and others examining neural substrates related to painful versus nonpainful stimuli during working memory tasks (Oshiro et al., 2007), no research has compared the two aspects of somatosensation when subjects actively encode and passively receive stimulation to uncover unique mechanisms for the encoding of pain. By using a delayed-discrimination task and fMRI, we investigated the neural mechanisms underlying the encoding of painful versus nonpainful stimuli. The inclusion of both stimulus types allowed a direct comparison and enabled us to identify the neural substrates relevant to pain. Moreover, an offset detection task under encoding and nonencoding conditions effectively controlled the influence of attention on the working memory encoding process (Vogel et al., 2005; Gazzaley and Nobre, 2012), which enabled us to identify encoding-related neural correlates in an unbiased way. We hypothesized that distinct somatosensation-processing brain regions, such as the thalamus, somatosensory cortex, and cingulate cortex (Preuschhof et al., 2006; Albanese et al., 2007; Kostopoulos et al., 2007; Oshiro et al., 2007), would be engaged in the encoding of painful and nonpainful stimulation. Given that pain is an event with a strong emotional component (Price, 2000), we further proposed that participants' emotions (as operationalized by their self-reported state anxiety) would modulate the activity in pain encoding-related circuits and emotion-related brain structures, especially the amygdala (Pessoa, 2008).

## Materials and Methods

### 

#### 

##### Subjects.

Twenty-three healthy right-handed participants who had never participated in studies using thermal or vibrotactile stimulation before were recruited for this study. The study protocol was approved by the University of Oxford Central University Research Ethics Committee, and informed consent was obtained from all participants before the experimental procedures. Three participants had to be excluded for the following reasons: one participant was insensitive to heat pain stimulation, one participant's images contained artifacts, and for one participant we were not able to collect the high-resolution T1 image needed for registration and normalization. Consequently, data from 20 participants (9 men and 11 women), 21–36 years of age (mean: 27.2 years), were analyzed. Before imaging, each participant's state and trait anxiety level as well as vigilance level to pain were assessed with the self-reported State-Trait Anxiety Inventory (STAI) (Spielberger et al., 1983) and Pain Vigilance and Awareness Questionnaire (PVAQ) (McCracken, 1997). To examine whether anxiety or attention to pain influenced the error rates, reaction time, and task difficulty, a split according to the median value of STAI scores and PVAQ scores was used to divide participants into two groups.

##### Stimuli.

Painful stimulation consisted of rapid ramping (30°C in 0.8 s) heat stimuli, which were delivered by two custom-built thermal resistors (surface size: 1.5 × 2 cm) (Wanigasekera et al., 2012). To efficiently titrate the stimulus pairs in the delayed-discrimination task in each participant before scanning, we chose vibrotactile frequency discrimination as a nonpain control condition, given that the human capacity for frequency discrimination in flutter is well characterized and can be easily manipulated (Mountcastle et al., 1990; Romo and Salinas, 2003). For vibrotactile stimulation, a piezo tactile stimulator (Dancer Design) with two piezoelectric stimulation probes containing built-in oscillators was used. Participants placed their left palm on the four stimulators, with the tips of the index and ring finger on the vibrotactile stimulator probes and the base of the two fingers on the thermal stimulators. All stimulators were MRI-compatible. To prevent displacement of stimulators during the experiment, the two fingertips were fixed on the vibrotactile stimulator heads with tape.

##### Behavioral and training sessions.

Before the fMRI experiment, each participant took part in three behavioral sessions to determine four stimulation temperatures and four vibration frequencies. We used the first two sessions to familiarize participants with the stimuli and determine the highest tolerable heat temperature for each participant. Stimuli in these sessions were of 4 s duration with 20 s interstimulus intervals. In the first session, participants received a series of ascending heat stimuli in steps of 1°C from 42°C to their highest tolerable temperature. In the second session, participants received a series of vibratory stimuli, the frequency of which ranged between 5 and 50 Hz in steps of 5 Hz, given that this range of frequency produces flutter sensations in humans (Romo et al., 1998). The third session contained alternating pain (42°C to the highest tolerable temperature) and vibrotactile (5–50 Hz) stimuli, with each stimulus presented twice and all stimuli presented in a random order. Throughout these sessions, stimuli of the same modality were delivered alternatively by the two stimulators, and participants were asked to rate the stimulus intensity immediately after each stimulus on a visual analog scale (VAS) with the descriptors “no pain”/“no vibration” and “very intense pain”/“very high frequency” as verbal anchors at the left and right ends of the scale, respectively. For each participant, two stimulus magnitudes (representing an individual perception boundary) equivalent to the 25th percentile (designated as Low) and the 75th percentile (designated as High) on the VAS were determined by linear interpolation using data obtained from the third session. Four pain stimulus magnitudes (Low pain stimulus = Low −0.5°C or 0.5°C; High pain stimulus = High −0.5°C or 0.5°C) and 4 vibration stimulus frequencies (Low vibration stimulus = Low −2.5 Hz or 2.5 Hz; High vibration stimulus = High −2.5 Hz or 2.5 Hz) were then chosen for each participant (see Table 1).

**Figure 1.**
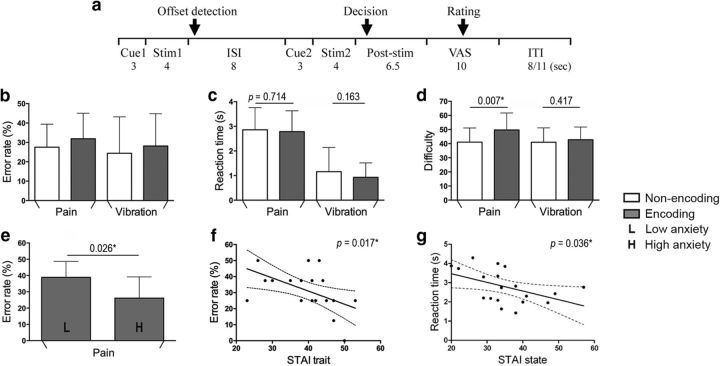
Experimental paradigm and behavioral results. ***a***, Red square (Cue1) represents an encoding trial, in which participants encoded the information of the first stimulus (Stim1) and, after an interstimulus interval, determined whether or not the second stimulus (Stim2), following another red square (Cue2), was of higher intensity (pain trials) or frequency (vibration trials) than Stim1. In a nonencoding trial indicated by a green square (Cue1), participants had to decide whether Stim2 had higher intensity or frequency than a predetermined stimulus magnitude (Cue2). In nonencoding trials, Cue2 consisted of a VAS showing the rating bar advanced to the 25th or 75th percentile, the corresponding stimulus intensity of which had been defined individually before the fMRI experiment. For all trials, participants were asked to detect the termination of Stim1 via button press and show their judgment as soon as they were confident in their decision after the offset of Stim2. After comparisons were made, participants used VAS to rate the task difficulty (for all trials), intensity of Stim2 (for nonencoding trials), or differences of stimuli (for encoding trials). The intertrial interval (ITI) was 8 or 11 s (for details, see Materials and Methods). ***b***, The error rates were significantly different from chance level (i.e., 50%) in all trial types (all *p* < 0.0001). ***c***, Participants did not show any significant differences in the reaction time to detect the offset of Stim1 between encoding and nonencoding trials, suggesting similar levels of attention. ***d***, For subjective task difficulty, encoding and nonencoding trials had significantly different levels of difficulty for pain trails, and there was a significant stimulus × task interaction. ***e***, Using a median split on state anxiety scores (assessed with the STAI) to divide participants into separate groups, the error rate was significantly different between low- and high-anxious participants in pain encoding trials. ***f***, ***g***, During the encoding of pain, trait and state anxiety were negatively correlated with the error rates and the response latency to detect the offset of Stim1, respectively. *p < 0.05. Error bars indicate SD.

Subsequently, participants performed a training session to familiarize themselves with the experimental procedures, which were identical to those used during the fMRI experiment. Each trial comprised two 4 s stimuli of the same modality, both of which were preceded by a 3 s cue period and separated by an 8 s delay (see Fig. 1*a*). A trial started with the first cue period during which a red or green square presented on the computer screen indicated an encoding or nonencoding trial, respectively. In an encoding trial, participants had to keep the first stimulus in mind and, after the delay and the second cue period consisting of the same red square, had to decide whether or not the second stimulus was of higher pain intensity or vibration frequency than the first one. In a nonencoding trial, participants were not required to remember the first stimulus but instead had to judge the intensity of the second stimulus. In such a nonencoding trial, the second cue consisted of a VAS showing the rating bar advanced to the 25th (Low) or 75th percentile (High) of the VAS, the corresponding stimulus intensity of which had been defined individually in the behavioral session (see above). Participants had to decide whether or not the magnitude of the following stimulus was higher than this perception boundary (chance level was 50%, as the stimulus could be 0.5°C higher or lower in the case of heat stimulation, or could be 2.5 Hz higher or lower in the case of vibratory stimulation; see above). Importantly, for both the first and the second stimuli, participants were trained to respond as fast as possible after each stimulus terminated: they had to signal the offset of the first stimulus by pressing any button on an MR-compatible button-box with their right hand, and press either the right middle finger (“yes”) or right index finger (“no”) once they were confident in their decision regarding the second stimulus (decision on encoding trials: higher than the first stimulus; decision on nonencoding trials: higher than the perception boundary). To make sure that participants had understood the task and performed correctly during the training session, all encoding trials contained one Low and one High stimulus presented in a random order, and the second stimulus in nonencoding trials was always very dissimilar to the preceding perception boundary. Participants had to perform a minimum of 10 pain and 10 vibration trials and were only qualified to start with the fMRI session if the last five trials were performed correctly. Finally, before imaging started and while participants lay in the scanner, we asked them to perform five additional practice trials for each stimulus type to ensure proper task performance during the fMRI experiment.

##### Experimental design.

The fMRI experiment (as described above) consisted of 32 trials and followed a 2 (pain vs vibration) × 2 (encoding vs nonencoding) factorial design, with 8 trials for each of the 4 trial types. Pain trials and vibration trials alternated over the course of a scanning session, and encoding and nonencoding trial types were presented in random order. For both encoding and nonencoding trials, two Low or two High stimuli comprised 50% of the trials, and 50% of the trials consisted of one Low and one High stimulus. The order of the two stimuli presented within each trial was counterbalanced and pseudo-randomized across trials. To examine whether individuals showed habituation or sensitization of responses to repeated somatosensory stimuli, participants were requested to rate the pain intensity or vibration frequency of the second stimulus after the decision task on a VAS in nonencoding trials (see Table 1). They were also required to rate task difficulty (on a VAS with the descriptors “easy” and “hard” as verbal anchors at the left and right ends of the scale) in all trials and the difference between the two stimuli (with “small” and “large” as verbal anchors at the ends of a VAS scale) in encoding trials. The intertrial interval was 8 or 11 s.

##### Statistical analysis.

Statistical analyses were performed using SPSS (IBM) and Prism (GraphPad Software). One-sample *t* tests were used to examine whether the memory task performance was significantly different from chance level (i.e., 50%) in each of the trial types. The main effects of stimulus and task type on the reaction time to detect the offset of the first stimulus as well as their interaction were analyzed by a repeated-measures ANOVA. Paired *t* tests were conducted to compare perceptual ratings and the reaction time between different tasks in pain or vibration trials. Pearson's correlation test was used to investigate the linear relationship between two variables.

##### fMRI data acquisition.

All images were acquired using a 3T scanner (Verio, Siemens). The participant's head was comfortably positioned inside a 32-channel head coil and padded with foam cushions to minimize head motion. Gradient-echo EPI was used to acquire BOLD data with the following parameters: a TR/TE of 2000/30 ms, a flip angle of 90°, a 64 × 64 matrix, a FOV of 192 × 192 mm, an acceleration factor of 2 (GRAPPA algorithm), and a slice thickness of 3.5 mm, resulting in a voxel size of 3 × 3 × 3.5 mm. In total, 36 horizontal slices without slice gap were obtained covering the entire brain. To correct possible geometric distortions of functional data, B0 field maps with identical FOV and matrix were acquired using a symmetrical-asymmetrical spin-echo sequence (TE = 30 ms, dwell time = 0.26 ms). In addition, a T1-weighted structural scan (1 × 1 × 1 mm) was acquired.

##### fMRI data analysis.

fMRI image processing and data analysis were performed using FMRI Expert Analysis Tool (FEAT), version 5.98, part of the Functional Magnetic Resonance Imaging of the Brain (FMRIB) Software Library (FSL; http://www.fmrib.ox.ac.uk/fsl/). Prestatistics processing of functional imaging data was performed in the following way: motion correction using MCFLIRT (Jenkinson et al., 2002), B0 unwarping using field maps, removal of nonbrain structures using Brain Extraction Tool (Smith, 2002), spatial smoothing using a Gaussian kernel with a 5 mm FWHM, and high-pass temporal filtering (cutoff: 100 s). Time-series autocorrelation correction was performed using FMRIB's Improved Linear Model (FILM) (Woolrich et al., 2001).

Each individual's first-level analysis was performed using a GLM, which modeled the fMRI time-series as a sequence of events convolved with a hemodynamic response function (gamma function with a SD of 3 s and a mean lag of 6 s). The current study mainly focused on the period of the first stimulus, which was categorized according to task and stimulus types (i.e., pain encoding trials, pain nonencoding trials, vibration encoding trials, and vibration nonencoding trials). Only neuroimaging results related to this period of the trial are presented (i.e., no data on delay and decision periods are presented). Other regressors included the periods of cue1, offset detection, delay, cue2, second stimulus, poststimulation and rating (see Fig. 1*a*). The intertrial interval was not modeled and served as the implicit baseline. fMRI data were coregistered to each participant's structural scan (T1-weighted) using a boundary-based registration procedure (Greve and Fischl, 2009). Subsequently, they were spatially normalized to the MNI-152 standard brain using an initial linear registration (FLIRT, FMRIB's Linear Image Registration Tool) (Jenkinson et al., 2002) and a following nonlinear registration (FNIRT, FMRIB's Non-Linear Image Registration Tool) (Andersson et al., 2007). For each participant, average parameter estimates in brain regions showing significant activation or psychophysical interaction (PPI) effects in PPI analyses (described below) were extracted from the peak voxel of a cluster using the fslmeants command implemented in FSL.

For stimulation-related activations, we first conducted a conjunction analysis across both types of stimulation to test where activations to pain and vibration overlap. Another conjunction analysis of “pain > baseline” and “pain > vibration” contrasts was performed to detect brain regions showing stronger responses to painful stimuli, and the conjunction of “vibration > baseline” and “vibration > pain” contrasts was analyzed to detect brain regions showing stronger responses to vibrotactile stimuli. To identify encoding-related activity, the “encoding > nonencoding” contrast was analyzed for each type of stimulus, and an interaction contrast (“encoding > nonencoding” × “pain > vibration”) was performed to investigate brain activations attributed to the encoding of pain. For these encoding-related contrasts, we conducted two further analyses. First, to examine whether pain encoding-related activity as well as the effect of anxiety were sensitive to pain intensity, we separated each of the four regressors during the period of the first stimulus into a high-magnitude and a low-magnitude regressor according to Table 1. Second, to investigate whether brain responses associated with successfully encoded conditions (i.e., correct trials) differed from those during unsuccessfully encoded conditions (i.e., error trials), we divided each regressor into a “successful” and an “unsuccessful” regressor according to participants' responses in another first-level GLM in FSL.

Because we hypothesized that working memory encoding-related brain activity was not necessarily spatially distinct from activations to somatosensory stimulation, we performed small-volume corrections in brain areas most commonly activated by pain (Duerden and Albanese, 2013) and vibration (Francis et al., 2000; Preuschhof et al., 2006; Albanese et al., 2009), which included the primary (SI) and secondary (SII) somatosensory cortices, thalamus, insula, and ACC bilaterally. We also included the bilateral hippocampi as a region of interest (ROI) due to its crucial role in mnemonic processes. Because we hypothesized a role of emotions in the encoding of painful stimuli, small-volume correction in bilateral amygdala was also performed to examine whether this brain region mediated emotional modulation during the encoding of pain. These regions were defined using the Harvard-Oxford Cortical and Subcortical Structural Atlas (thalamus, insula, ACC, amygdala, hippocampus) and the Juelich Histological Atlas (SI, SII) thresholded at 50% probability (see Table 3). Considering the presence of a clear somatotopic organization, the ROI for the bilateral SI was defined as the intersection between the mask in the Juelich Histological Atlas and a 10 mm radius sphere centered at MNI coordinates (±56, −28, 52), as in a previous fMRI study applying somatic stimuli to the finger (Preuschhof et al., 2006). To adequately control false-positives (Eklund et al., 2016), statistical testing for both at the whole-brain and ROI level in the current study was based on nonparametric permutation testing (5000 permutations) implemented in the Randomize function of FSL (Nichols and Holmes, 2002), using a cluster-defining threshold of *p* < 0.001 and a cluster significance threshold of *p* < 0.05 (familywise error-corrected). The correction for multiple comparisons was restricted within a whole-brain mask (232,261 voxels) that was obtained from the analysis of average group statistical maps in FEAT, or within a priori ROI masks (for small-volume corrections; see Table 3). Anatomical locations of activation peaks within significant clusters were determined by reference to the Harvard-Oxford and Juelich atlases (Desikan et al., 2006; Eickhoff et al., 2007), and the coordinates with the highest probability of belonging to a distinct brain region were reported as the local maxima.

##### Psychophysiological interaction analysis.

A PPI means that the interregional functional connectivity (i.e., temporal correlation between BOLD signals of different brain areas) significantly changes with the experimental context (Friston et al., 1997; O'Reilly et al., 2012). We performed PPI analyses to search for brain regions whose functional connectivity with the bilateral thalamus, the right ACC, and the left SI is influenced by different encoding conditions (i.e., encoding and nonencoding trials). We selected these three regions for PPI analyses because their activity was related to the encoding of painful or nonpainful stimulation (see Fig. 3). We used anatomical masks of the thalamus, ACC, and SI from above-described atlas (again thresholded at 50% probability) as seed regions and extracted subject-specific average time courses. The PPI regressor was obtained from the product of a vector representing the contrast of tasks (contrast weight of −1 for nonencoding tasks and 1 for encoding tasks; psychological regressor) and the mean time courses across all voxels within a seed region (physiological regressor). The GLM for the PPI analyses in each participant thus included the psychological regressor, the physiological regressor, and the PPI regressor. Individual PPI maps were entered into a group-level mixed-effects analysis to identify functional connectivity changes related to encoding versus nonencoding of pain and vibration stimulation at the group level (using the same statistical thresholding procedures as described above).

## Results

### Behavioral results

On average, painful stimuli elicited moderate pain (mean ± SD, VAS rating = 45.1 ± 12.1; Table 1). There was no significant difference in pain intensity ratings between the very first (mean ± SD, high pain = 72.6 ± 19.1, low pain = 29.6 ± 22.6) and the very last (high pain = 62.8 ± 25.3, low pain = 24.2 ± 13.9) stimulus of either high pain (*t* = 1.326, *p* = 0.201) or low pain (*t* = 1.148, *p* = 0.264), suggesting that sensitization or habituation did not occur across the repeated painful stimuli in our paradigm.

**Table 1.** Stimulus intensity and perceptual ratings on VAS*^a^*

	Pain				Vibration			
	Low −0.5°C	Low +0.5°C	High −0.5°C	High +0.5°C	Low −2.5 Hz	Low +2.5 Hz	High −2.5 Hz	High +2.5 Hz
Intensity	44.8	45.3	51.2	51.7	15.9	18.4	41.7	44.2
	(2.2) °C	(2.2) °C	(1.9) °C	(1.9) °C	(4.5) Hz	(4.5) Hz	(6.7) Hz	(6.7) Hz
Rating	28.5	28.8	63.7	67.4	22.3	27.0	72.5	75.6
	(12.2)	(18.9)	(17.9)	(17.5)	(8.4)	(10.4)	(11.8)	(13.3)

*^a^*Values are mean (SD). Low and High indicate the 25th percentile and the 75th percentile stimulus magnitudes, respectively, calculated by linear interpolation in each participant.

For both painful heat and nonpainful vibrotactile stimulation, error rates were significantly lower than chance (i.e., 50%) in all trial types (pain nonencoding: 27.5%; pain encoding: 31.9%; vibration nonencoding: 24.4%; vibration encoding: 28.1%; all *p* < 0.0001; Fig. 1*b*). These findings indicate that participants successfully encoded the information of the first stimulus for later comparisons during encoding trials, and concentrated on the categorization of the second stimulus during nonencoding trials. There was no significant main effect of task (encoding vs nonencoding, *F*_(1,19)_ = 1.507, *p* = 0.235), no significant main effect of stimulus (pain vs vibration, *F*_(1,19)_ = 1.292, *p* = 0.270), and no significant stimulus × task interaction (*F*_(1,19)_ = 0.011, *p* = 0.918).

For the reaction time to detect stimulus offset, there was a main effect of the stimulus (pain vs vibration, *F*_(1,19)_ = 83.000, *p* < 0.001), with longer response latency for pain (2820 ± 864 ms) compared with vibration (1043 ± 807 ms), which is likely due to the prolonged time for the skin temperature to return to the baseline after a contact heat stimulus (Granot et al., 2006). There was no significant main effect of task (encoding vs nonencoding, *F*_(1,19)_ = 1.448, *p* = 0.244), no effect of task in pain trials (*p* = 0.714) or vibration trials (*p* = 0.163), and no significant stimulus × task interaction (*F*_(1,19)_ = 0.337, *p* = 0.568; Fig. 1*c*), which is very important because it indicates that participants paid comparable levels of attention to the first stimulus in both encoding and nonencoding trials.

Regarding subjective task difficulty, there was a significant main effect of task (encoding vs nonencoding, *F*_(1,19)_ = 7.538, *p* = 0.013) but no significant effect of stimulus (pain vs vibration, *F*_(1,19)_ = 1.640, *p* = 0.216). The effect of task was also significant in pain trials (*p* = 0.007) but not vibration trials (*p* = 0.417). There was a significant stimulus × task interaction (*F*_(1,19)_ = 4.504, *p* = 0.047; Fig. 1*d*), with greater difficulty ratings for the encoding condition when participants processed painful stimuli. For both the error rate and task difficulty, comparing two painful stimuli with similar intensities (i.e., encoding trials consisting of either two Low or two High stimuli; Table 1) was significantly more error-prone and more difficult than comparing two painful stimuli with dissimilar intensities (i.e., encoding trials consisting of one Low and one High pain stimulus; error rate: 50.0 ± 16.2% vs 13.8 ± 17.2%, *p* < 0.0001; task difficulty: 59.1 ± 18.9 vs 40.3 ± 12.8, *p* = 0.0009).

### Brain activation related to painful and nonpainful stimulation

**Figure 2.**
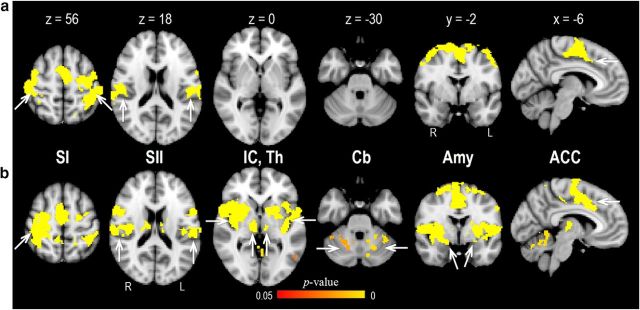
Brain activation to pain and vibration. ***a***, A conjunction analysis (“pain > baseline” and “vibration > baseline” contrasts) revealed several brain regions responsive to both types of stimulation, including SI and SII, and ACC. ***b***, A conjunction analysis (“pain > baseline” and “pain > vibration” contrasts) showed that painful stimuli evoked significantly stronger activation in a wide array of brain regions, including SI, SII, insular cortex (IC), thalamus (Th), cerebellum (Cb), amygdala (Amy), and ACC. ***a***, ***b***, Clusters showing significant activations are reported at a threshold of *p* < 0.05, corrected for multiple comparisons (for details, see Materials and Methods; Table 2).

Consistent with previous research (Coghill et al., 1994; Tseng et al., 2010), activations to painful and nonpainful stimulation overlapped in several brain areas, including SI, SII, ACC, supplementary motor area, precentral gyrus, PFC, and posterior parietal cortex (whole-brain correction; Fig. 2*a*; Table 2). Comparison of pain trials with vibration trials during the period of the first stimulus showed enhanced responses in regions identified previously in pain imaging studies, such as SI, SII, thalamus, insular cortex, ACC, PFC, and amygdala (Peyron et al., 2000) (whole-brain correction; Fig. 2*b*; Table 2). On the contrary, there were no brain regions that exhibited higher BOLD signals to vibration compared with pain.

**Table 2.** Conjunction analysis of activation patterns*^a^*

Brain area	Side	P and V					P and (P > V)				
		*x*	*y*	*z*	*p*	Cluster no. (size)	*x*	*y*	*z*	*p*	Cluster no. (size)
Thalamus	R	—	—	—	—	—	16	−22	−2	<0.001	1 (12877)
	L	—	—	—	—	—	−6	−22	−2	<0.001	2 (253)
SI	R	46	−36	56	<0.001	1 (9023)	22	−42	56	<0.001	1
	L	−28	−46	56	<0.001	1	—	—	—	—	—
SII	R	46	−28	18	<0.001	2 (692)	50	−28	18	<0.001	1
	L	−46	−30	18	<0.001	1	−52	−30	18	<0.001	3 (5623)
IC	R	—	—	—	—	—	36	−18	−4	<0.001	1
	L	—	—	—	—	—	−42	4	−2	<0.001	3
ACC	R	—	—	—	—	—	2	2	36	<0.001	1
	L	−4	6	40	<0.001	1	−4	−4	38	<0.001	1
PCC	R	—	—	—	—	—	14	−30	38	<0.001	1
							4	−38	24	<0.001	4 (102)
	L	—	—	—	—	—	−14	−32	38	<0.001	5 (114)
							−2	−36	24	<0.001	4
SFG	R	16	10	62	<0.001	1					
	L	—	—	—	—	—	−12	−6	62	<0.001	1
MFG	R	—	—	—	—	—	48	32	28	0.034	6 (201)
IFG	R	—	—	—	—	—	44	44	8	0.024	7 (25)
SMA	R	2	−12	52	<0.001	1					
	L	—	—	—	—	—	−12	−6	62	<0.001	1
PCG	R	22	−14	58	<0.001	1	30	−10	50	<0.001	1
	L	−36	−20	52	<0.001	1	−22	−12	48	<0.001	1
PPC	R	56	−32	54	<0.001	1	46	−26	36	<0.001	1
	L	−16	−64	54	<0.001	1	−48	−42	34	<0.001	3
Amygdala	R	—	—	—	—	—	26	−4	−14	<0.001	1
	L	—	—	—	—	—	−22	−2	−12	<0.001	3
Cerebellum	R	—	—	—	—	—	0	−68	−22	0.007	8 (911)
							36	−46	−36	0.018	9 (183)
	L	—	—	—	—	—	−8	−64	−24	0.007	8
							44	−50	−30	0.018	10 (13)
Putamen	L	—	—	—	—	—	−34	−4	−8	<0.001	3
MTG	L	—	—	—	—	—	−52	−60	−2	0.027	11 (92)
VC	R	—	—	—	—	—	58	−60	−6	<0.001	12 (5)
	L	—	—	—	—	—	−2	−74	6	0.007	13 (2)

*^a^*The peak MNI *x*, *y*, *z* coordinates (mm), corrected *p* values, and suprathreshold cluster number (sizes in voxels) of whole-brain corrections. The conjunction analysis [P (pain > baseline) and V (vibration > baseline) contrasts] searches for commonly activated regions for both stimulations, whereas the conjunction analysis [P and (P > V)] showed brain regions with both significant activation to pain and enhanced activation to pain compared with vibration. Activations are thresholded at a threshold of *p* < 0.05, corrected for multiple comparisons. R, Right; L, left; IC, insular cortex; PCC, posterior cingulate cortex; SFG, superior frontal gyrus; MFG, middle frontal gyrus; IFG, inferior frontal gyrus; SMA, supplementary motor area; PCG, precentral gyrus; PPC, posterior parietal cortex; MTG, middle temporal gyrus; VC, visual cortex.

### Brain activation related to encoding of painful and nonpainful stimulation

**Figure 3.**
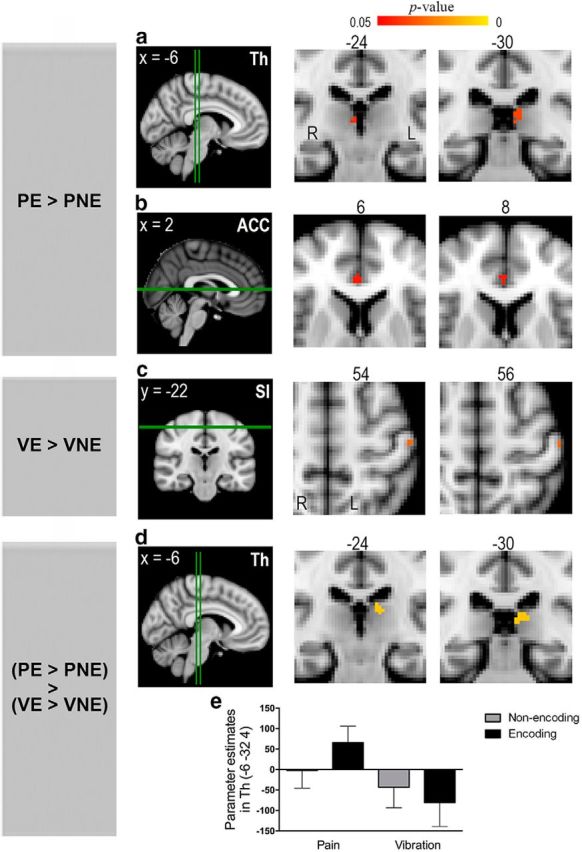
Dissociation of brain regions related to the encoding of painful versus nonpainful stimulation. This figure illustrates the results of small-volume corrections in ROI (for details, see Table 3). Leftmost column represents the individual contrast. E, Encoding; NE, nonencoding; P, pain; V, vibration. Green lines indicate the level of the coronal and axial sections. ***a***, ***b***, The bilateral thalamus (Th) and the right ACC exhibited enhanced activity during the encoding of pain. ***c***, The left SI showed increased activity during the encoding of vibration. ***d***, An interaction contrast revealed significant activation in the left thalamus. ***a–d***, Clusters showing significant activations are reported at a threshold of *p* < 0.05, corrected for multiple comparisons (for details, see Materials and Methods). ***e***, The parameter estimates (mean ± SEM) were extracted from the peak voxel within the activated thalamic cluster in ***d***.

To investigate whether distinct brain activity patterns were associated with the encoding of pain compared with nonpainful stimulation, we examined BOLD responses during the encoding of pain and vibration (see Materials and Methods). As summarized in Table 3, some stimulation-related brain regions exhibited enhanced responses during encoding. Notably, the neural encoding of painful versus nonpainful stimulation was dissociable. Compared with the nonencoding trials, activity of bilateral midline and mediodorsal portions of the thalamus (right thalamus: *p* = 0.041; left thalamus: *p* = 0.036; Fig. 3*a*) and the rostral portion of the right ACC (*p* = 0.044; Fig. 3*b*) was enhanced during the encoding of pain; this effect was not seen for vibration. A further interaction analysis contrasting the effect of stimulus type (pain vs vibration) within encoding compared with the nonencoding condition demonstrated significantly different thalamic responsivity associated with pain (i.e., a significant interaction at *p* = 0.012; Fig. 3*d*,*e*). These results held when individual ratings of task difficulty were included as a covariate in the group analyses. Moreover, the observed thalamic activation (Fig. 3*d*) was significantly higher during correct (successful) trials than during error (unsuccessful) trials (mean ± SD, parameter estimates: correct vs error trials = 48.71 ± 83.52 vs 12.07 ± 61.70, *p* = 0.041), although small-volume corrections did not reveal significant clusters in pain encoding contrast (i.e., “pain encoding > pain nonencoding”) between correct and error trials, possibly due to a lack of power brought about by the small number of trials. Also, these pain encoding-related activations were not significantly different between high pain and low pain trials. In contrast, encoding of vibration (Fig. 3*c*) entailed an increased response in left SI (*p* = 0.030); this effect was not seen for pain. The difference in left SI activation between encoding and nonencoding trials for vibration was not significantly higher than the difference between encoding and nonencoding trials for pain. The amygdala was activated in pain trials (pain encoding trials: *p* = 0.003 and *p* = 0.037 for the right and left amygdala, respectively; pain nonencoding trials: *p* = 0.013 for the right amygdala), but the activity was not significantly different between task types (encoding vs nonencoding). The bilateral hippocampi, the core region related to long-term memory encoding (Dolan and Fletcher, 1997), were not significantly activated for either pain or vibration trials.

**Table 3.** Encoding-related activation in a priori ROI*^a^*

ROI (voxels)		PE	PNE	VE	VNE	PE > PNE	VE > VNE	(PE > PNE) > (VE>VNE)
Thalamus (2264)	R	16/−24/−2	10/−24/−2	—	—	6/−24/4	—	—
		(0.001; 647)	(0.001; 412)			(0.041; 22)		
	L	−6/−22/−2	−6/−22/−2	—	—	−6/−30/4	—	−6/−32/4
		(0.002; 400)	(0.002; 214)			(0.036; 25)		(0.012; 65)
SI (275)	R	48/−28/46	48/−28/46	52/−24/46	48/−28/46	—	—	—
		(<0.001; 111)	(0.002; 111)	(0.001; 98)	(0.001; 108)			
	L	−50/−28/44	−50/−28/44	−50/−28/44	−50/−28/44	—	−52/−22/54	—
		(<0.001; 160)	(<0.001; 159)	(<0.001; 164)	(<0.001; 164)		(0.030; 5)	
SII (1431)	R	50/−26/16	50/−26/16	52/−24/16	56/−20/14	—	—	—
		(<0.001; 625)	(<0.001; 566)	(0.003; 150)	(0.001; 200)			
	L	−64/−26/18	−64/−26/18	−48/−20/14	−48/−28/16	—	—	—
		(<0.001; 488)	(<0.001; 433)	(0.004; 115)	(0.001; 191)			
IC (1080)	R	40/0/−14	42/6/0	—	—	—	—	—
		(<0.001; 400)	(<0.001; 302)					
	L	−42/8/−6	−38/−4/−12	—	—	—	—	—
		(<0.001; 398)	(<0.001; 389)					
ACC (1531)	R	2/12/32	2/10/30	—	—	4/34/6	—	—
		(<0.001; 399)	(<0.001; 396)			(0.044; 14)		
	L	−2/14/30	−2/0/36	—	—	—	—	—
		(<0.001; 399)	(<0.001; 396)					
Amygdala (852)	R	28/−4/−14	16/−8/−18	—	—	—	—	—
		(0.003; 45)	(0.013; 29)					
	L	−24/−4/−12	—	—	—	—	—	—
		(0.037; 14)						
Hippocampus (1055)	R	—	—	—	—	—	—	—
	L	—	—	—	—	—	—	—

*^a^*Values are MNI coordinates (*x*, *y*, *z*) (*p* value; cluster size). The peak MNI *x*, *y*, *z* coordinates (mm), corrected *p* values, and suprathreshold cluster sizes (in voxels) of small-volume corrections in bilateral masks. E, Encoding; NE, nonencoding; P, pain; V, vibration; —, no significant activation; R, right; L, left; ACC, anterior cingulate cortex; IC, insular cortex.

### Functional connectivity underlying the encoding of pain

Next, we investigated changes in brain connectivity that underlie the process of encoding pain. To that end, we performed PPI analyses to identify regions whose functional connectivity with the seed area changes as a function of the psychological context (i.e., the four experimental conditions). We extracted the BOLD time-series in encoding-related brain regions (i.e., the bilateral thalamus, the right ACC, and the left SI; Fig. 3) to search for coactivated brain regions. Given that the affective dimension is an essential feature of pain (Price, 2000), we assumed that brain regions integrating emotion and cognition would be recruited as people executed cognitive operations to convert a painful experience into a momentarily memorable construct.

**Figure 4.**
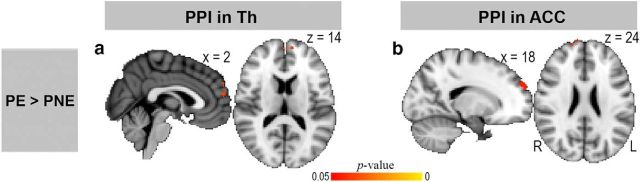
Functional connectivity for the encoding of pain. This figure shows the results of whole-brain PPI analyses. Leftmost column represents the individual contrast. PE, Pain encoding; PNE, pain nonencoding. ***a***, ***b***, Functional connectivities from the bilateral thalamus (Th) and the right ACC to the mPFC were increased during pain encoding. Clusters showing significant activations are reported at a threshold of *p* < 0.05, corrected for multiple comparisons (for details, see Materials and Methods).

The results of our PPI analyses (Fig. 4) demonstrated that, across the entire brain, the mPFC was the only brain region showing enhanced functional coupling with the thalamus (*p* = 0.034; peak MNI coordinates: 2/58/14; cluster size: 348 voxels; Fig. 4*a*) and ACC (*p* = 0.037; peak MNI coordinates: 18/60/24; cluster size: 337 voxels; Fig. 4*b*) during the pain-encoding trials compared with pain nonencoding trials. By contrast, no brain areas showed significant connectivity changes with the three seed regions during the encoding of vibration.

To examine whether the revealed pain encoding-related thalamic activity (Fig. 3) and thalamic-mPFC coupling (Fig. 4) were associated with the behavioral measures, these fMRI data were correlated against a participant's perceived task difficulty, error rates, and response latency of offset detection during pain encoding trials. None of these analyses yielded a significant correlation (all *p* > 0.295).

### The influence of interindividual differences in anxiety during the encoding of pain

Given that the processing of pain is subject to different emotional and cognitive states across individuals (Rhudy and Meagher, 2000; Villemure et al., 2003; Rainville et al., 2005), we further examined whether pain encoding behavior as well as associated brain responses were biased by individual affectivity and attention to pain.

Intriguingly, during pain encoding trials, participants with high state anxiety levels (scores ≥ 34, *n* = 11) showed a significantly lower error rate compared with those with low state anxiety levels (scores ≤ 33, *n* = 9; *p* = 0.026, two-tailed; Fig. 1*e*); very similar findings were obtained when using participants' trait anxiety levels (high vs low trait anxiety scores, *p* = 0.026, two-tailed), although this is not too surprising because there was a modest correlation between state and trait scores (*r* = 0.435, *p* = 0.055), although the interaction contrast for this effect failed to reach significance (condition [pain encoding, pain nonencoding] × group [high anxiety, low anxiety] interaction: *p* = 0.140). The state anxiety score was inversely related to the error rate during pain encoding trials, but this correlation did not reach statistical significance (*r* = −0.310, *p* = 0.183). With respect to trait anxiety, there was a negative correlation between trait anxiety and error rates (*r* = −0.527, *p* = 0.017; Fig. 1*f*), but this was not specific for only high pain or low pain trials. Moreover, error rates were neither associated with state or trait anxiety in pain nonencoding trials and both types of vibration trials (all *p* > 0.37), nor did they differ between different levels of attention toward pain as assessed with PVAQ (all *p* > 0.16).

Similar to the results on error rates, only in pain encoding trials, but not the other three conditions, participants with high state anxiety scores showed a significantly faster reaction time to detect the offset of the first stimulus compared with those with low state anxiety scores (*p* = 0.031, two-tailed; all other *p* > 0.08), and the reaction time for offset detection was indeed inversely correlated with individual state anxiety (*r* = −0.471, *p* = 0.036; Fig. 1*g*), although, again, the interaction effect failed to reach significance (condition [pain encoding, pain nonencoding] × group [high anxiety, low anxiety] interaction: *p* = 0.845). Here, state anxiety significantly reduced the reaction time during high pain stimuli (high-anxiety group vs low-anxiety group: 2.68 vs 3.71 s; *p* = 0.015, two-tailed) but not during low pain stimuli (*p* = 0.129), and the above-reported inverse relationship between the reaction time and individual state anxiety only existed during high pain stimuli (*r* = −0.485, *p* = 0.030) but not during low pain stimuli (*p* = 0.090). The response latency was not affected by different levels of trait anxiety or PVAQ scores in any trial types (all *p* > 0.13). For all experimental conditions, task difficulty was not significantly different between different degrees of state anxiety, trait anxiety, or PVAQ scores (all *p* > 0.28). Together, these results suggest that interindividual differences in state and trait anxiety (but not attention to pain) did relate to pain encoding-related behavior, with more anxious participants performing better on pain-encoding trials and reacting faster to detect the termination of painful stimuli.

**Figure 5.**
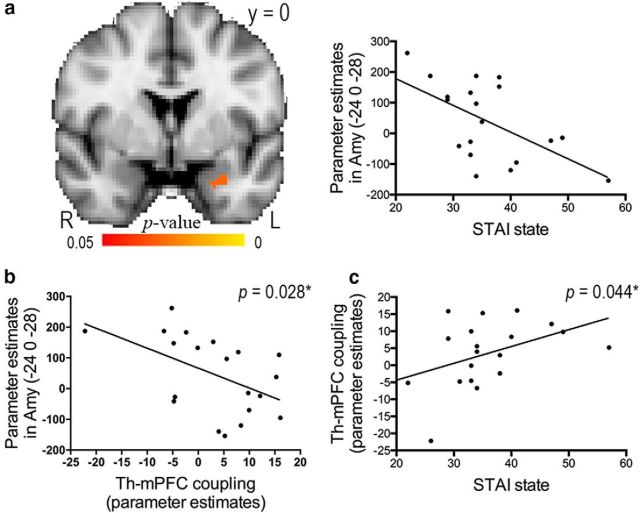
Correlations among individual state anxiety, brain activation, and functional connectivity during the encoding of pain. ***a***, This figure illustrates the results of small-volume corrections in the bilateral amygdala. Responsivity in the left amygdala (Amy) to pain was inversely correlated to individual state anxiety assessed with the STAI. The scatter plot illustrates the relationship between amygdala activation (parameter estimates extracted from the peak voxel in ***a***) and state anxiety. ***b***, ***c***, The strength of functional connectivity between the thalamus (Th) and mPFC (parameter estimates extracted from the peak voxel in Fig. 4*a*) was negatively correlated with left amygdala activity (*r* = −0.508, *p* = 0.028) and positively correlated with individual state anxiety (*r* = 0.455, *p* = 0.044).

These behavioral findings support the notion that emotions bias humans to act differently in response to threatening stimuli, such as pain (Ploner et al., 2010; Wiech et al., 2010). To explore whether and how state and trait anxiety modulated emotion- and/or pain encoding-related brain responses, we conducted an intersubject linear regression analysis of our fMRI data using the self-reported anxiety level as a covariate of interest, and examined the relationships among anxiety levels, anxiety-related brain activity, and the neural correlates of pain encoding (Figs. 3, 4). Importantly, we found that neural activity in the left amygdala was negatively correlated with state anxiety during pain encoding condition (*p* = 0.028; peak MNI coordinates: −24/0/−28; cluster size: 19 voxels; Fig. 5*a*) but not during pain nonencoding, vibration encoding, or vibration nonencoding trials. This relationship was only significant in high pain encoding trials (*p* = 0.024; peak MNI coordinates: −24/−2/−26; cluster size: 21 voxels) but not in low pain encoding trials. Both the amygdala activation and state anxiety were further correlated with the extent of coupling between thalamus and mPFC described in Figure 4*a* (*p* = 0.028 and *p* = 0.044, respectively; Fig. 5*b*,*c*). Moreover, there was a trend toward a positive correlation between individual trait anxiety and pain encoding-related thalamic activity described in Figure 3*a* (*r* = 0.436, *p* = 0.055). Indeed, this relationship was significant when participants encoded high pain stimuli (*r* = 0.592, *p* = 0.006). None of the correlations in Figure 5 was significant when participants processed pain in a nonencoding condition or processed vibration in an encoding or nonencoding condition.

## Discussion

In this study, we found that SI was involved in the encoding of vibrotactile sensation. By contrast, medial thalamic and rostral ACC activity was associated with the encoding of pain, which was furthermore associated with enhanced functional connectivity between thalamus and mPFC. Pain encoding-related behavior was modulated by participants' anxiety, which was mirrored in the orchestration of the medial thalamus, mPFC, and amygdala, especially during the encoding of high pain. Our findings suggest that the encoding of pain engages distinct neural mechanisms and that these mechanisms are shaped by an individual's emotional state.

### Working memory encoding of vibrotactile stimulation

The identification of SI to encode the frequency of vibration is consistent with previous research in which the firing rate of SI neurons codes for flutter frequency (Salinas et al., 2000). Although previous neuroimaging findings had reported increased SI activity during the encoding of vibrotactile stimulation (Preuschhof et al., 2006; Kostopoulos et al., 2007; Albanese et al., 2009), this phenomenon could possibly be attributed to the influence of attention because attention levels were likely unequal between tasks in these studies. It has been well documented that attention substantially influences working memory at multiple processing stages, including the encoding process (Vogel et al., 2005; Gazzaley and Nobre, 2012). Tasks requiring more attention are associated with enhanced SI activity (Staines et al., 2002). In our study, the reaction times to detect stimulus offset between encoding and nonencoding tasks were not significantly different, which suggests comparable levels of attention. Our paradigm thus allows the elucidation of the neural mechanisms for the encoding process without the potential confounding effects of attention.

Intriguingly, although we demonstrated that bilateral SI responded to vibrotactile stimulation, only the ipsilateral SI participated in the encoding process. Indeed, previous studies have documented the response in ipsilateral SI to innocuous somatosensory stimulation, possibly through uncrossed ascending tracts or transcallosal connections (Schnitzler et al., 1995; Korvenoja et al., 1999). In contrast to a well-characterized function of its contralateral counterpart in processing sensory-discriminative features (Nakamura et al., 1998; Stippich et al., 1999), the significance of the ipsilateral SI in somatosensory processing remains unclear. Nevertheless, evidence points to a role of ipsilateral SI beyond perception (Noachtar et al., 1997), and its activity has been shown to robustly vary across different cognitive states (Staines et al., 2002). The enhanced activation in ipsilateral SI during the encoding of vibrotactile stimulation, as revealed in the current study, provides an explanation for its functional significance in the processing of somatosensory inputs in humans.

### Working memory encoding of pain

In the current study, the engagement of the rostral ACC in pain encoding not only resonates with the observation that this brain area participates in the processing of threat-related stimuli (Bishop et al., 2004), but extends the role of ACC in pain processing given the rostral ACC has been implicated in the emotional processing of both acute pain (Vogt, 2005) and chronic pain (Qu et al., 2011). As for the medial thalamus, we show that the medial thalamus is particularly associated with pain encoding, as evidenced by the interaction contrasts. This structure contains many nociceptive-specific neurons (Craig, 1987) and has been implicated in mediating the affective-motivational aspects of pain (Price, 2000; Dostrovsky and Craig, 2013). Moreover, evidence suggests that the medial thalamus is involved in a diversity of cognitive functions, including attentional modulation of nociceptive processing (Bushnell and Duncan, 1989) and executive control of working memory (Floresco et al., 1999). In humans, neuronal loss and reduced activation in the medial thalamus have been related to cognitive impairment (Popken et al., 2000).

Our PPI analyses further revealed that both the thalamus and ACC showed significantly enhanced connectivity with the mPFC during pain encoding. With increased activity at rest (Raichle et al., 2001), the mPFC has been proposed to subserve working memory processes (Ramnani and Owen, 2004). A role for the mPFC in regulating emotion and cognition was further supported by the presence of cognitive dysfunction in subjects with mPFC lesions (Damasio and Van Hoesen, 1983) and abnormal mPFC activity in depressed patients (Yoshimura et al., 2010). Anatomically, both medial thalamus and the ACC are reciprocally interconnected with mPFC (Ray and Price, 1992; Carmichael and Price, 1995). Evidence suggests that the medial thalamus acts as an interface between the mPFC and hippocampus to subserve successful encoding (Aggleton and Brown, 2006), and reduced connectivity between the medial thalamus and mPFC has been associated with cognitive deficits in humans (Woodward et al., 2012). In combination with the role of mPFC in emotional regulation (Pessoa, 2008) as well as its interconnections with association cortices and limbic structures (Pandya and Yeterian, 1990), one might speculate that a medial-thalamic driven activation of the mPFC is related to incorporating the affective components of pain into working memory encoding processes. Based on our data, this encoding process is unlikely to be driven by self-monitoring or attention, given that (1) the observed pain encoding-related activation in the medial thalamus and functional connectivity between thalamus and mPFC were not associated with perceived task difficulty, task performance, and response latency, and (2) participants' attention to pain did not bias task performance. Interactions between amygdala, which is engaged in encoding emotionally arousing stimuli from the environment (Pessoa, 2008), and hippocampus, which is critical substrate for the encoding of long-term emotional memory (Richardson et al., 2004), were not discernible in our working memory encoding tasks. Thus, we propose that there exists a distinct neural stream in the human brain to subserve the working memory encoding of pain, and the emotional part of pain experience receives preferred processing when pain needs to be transformed into a maintainable construct.

### Individual differences in anxiety

Finally, we observed that anxiety not only enhanced task performance on pain encoding trials but modulated brain responses associated with pain encoding, especially during the encoding of high pain. The effect of trait anxiety was directly mirrored in the positive relationship between trait anxiety and activation in the medial thalamus during pain encoding, whereas state anxiety inversely predicted amygdala activity, which was further negatively correlated with the degree of thalamic-mPFC coupling. The functional interplay among medial thalamus, mPFC, and amygdala during pain encoding is in accordance with the neural circuitry involved in emotional learning in which both medial thalamus and mPFC project to amygdala to modulate its activity (McNally et al., 2011). Given the above-described enhanced medial thalamic activity during pain encoding as well as reciprocal excitatory connections between the medial thalamus and mPFC (Ray et al., 1992; Di Prisco and Vertes, 2006), heightened thalamic-mPFC coupling in anxious participants would translate into enhanced mPFC activity, which subsequently pertains to reduced amygdala activity based on an inhibitory effect of the mPFC on the responsiveness of the amygdala (Quirk et al., 2003). Because amygdalar neurons project to the hypothalamus, basal forebrain, and periaqueductal gray to modulate the behavioral responses to threat of aversive stimuli (LeDoux et al., 1988), the inverse correlation between the amygdala reactivity and state anxiety levels might be explained by a central coping strategy that attenuates the perceived distress (Petrovic et al., 2004). Although the orchestration of thalamus, mPFC, and amygdala has been generally implicated in implicit learning and remembering of fearful events (McNally et al., 2011), we suspect that this system might implement a cognitive mechanism that is especially prominent in more anxious subjects during the encoding of an aversive stimulus and thus results in more efficient performance (i.e., lower error rates and faster reaction times). Future studies will be needed to confirm whether the role of this system extends beyond the implicit learning of aversive stimuli, and establish whether these neural mechanisms are indeed pain-specific or might be better described in the broader context of salience (Forkmann et al., 2013).

The results of the current study should be interpreted with a limitation that both correct and error trials were collapsed together. The inclusion of different conditions in a single experiment allowed us to directly examine the neural substrates highly relevant to pain encoding. Nevertheless, the complexity of the experimental design and limited trial repetitions preclude further statistical comparisons between correct and error trials. Future studies comparing the successfully encoded versus the unsuccessfully encoded events would disentangle the brain networks related to the efficient encoding of noxious stimuli. Another caveat is that we could not provide evidence that the correlations between anxiety, amygdala activation, and thalamic-mPFC coupling were significantly increased during pain encoding. However, we have demonstrated that the behavioral and neural effects of anxiety only existed when participants encoded pain but not during pain nonencoding, vibration encoding, or vibration nonencoding trials. Further research should try to clarify whether anxiety specifically modulates pain encoding behavior.

In conclusion, the present study reveals that the working memory encoding of noxious stimuli relies on neural mechanisms that are distinct from those for the encoding of innocuous stimuli. Individual anxiety levels seem to have a strong impact on these mechanisms that allow us to convert the sensation of pain into a briefly maintainable construct.
